# Impact of split application of potassium fertilizer on yield, quality, and economic benefits of winter wheat

**DOI:** 10.3389/fpls.2026.1827899

**Published:** 2026-05-13

**Authors:** Shengyan Pang, Muhammad Fraz Ali, Xin Wang, Yongbing Wang, Xiaotian Ren, Tahir Shah, Xiang Lin, Dong Wang

**Affiliations:** 1 State Key Laboratory of Crop Stress Resistance and High-Efficiency Production, College of Agronomy, Northwest A & F University, Yangling, Shaanxi, China; 2 College of Natural Resources and Environment, Northwest A&F University, Yangling, Shaanxi, China; 3 Anhui Science and Technology University, Anhui, Fengyang, China

**Keywords:** grain quality, Huang-Huai-Hai Region, potassium management, potassium use efficiency, split application, winter wheat, yield components

## Abstract

**Background:**

Potassium (K) is essential for winter wheat growth, yield formation, and grain quality; however, it is commonly applied as a single basal dose in the Huang-Huai-Hai region despite large spatial variability in soil K availability, leading to inefficient utilization.

**Aims:**

This study aimed to evaluate K application strategies across contrasting regions and identify an optimal, site-specific K management approach.

**Methods:**

Field experiments were conducted during 2021–2022 and 2022–2023 at three sites with contrasting soil K levels. A two-factor randomized block design with three replicates was used, including two K rates (K1: 120 and K2: 60kg K_2_O ha^-1^) and five application methods: no K (T1), single basal (T2), two-split (T3: 50% basal + 50% jointing), three-split (T4: 20% basal + 30% jointing + 50% anthesis), and single controlled-release basal (T5). The strong-gluten winter wheat cultivar ‘Weilong 169’ was used.

**Results:**

Basal and controlled-release treatments (T2 and T5) increased the available K in the 0–60-cm soil layer during early growth, while split application (T3 and T4) improved the K availability in the surface layer (0–30 cm) during later stages. The three-split application (T4) consistently improved K accumulation, 1, 000-grain weight, and grain yield across all sites. In high-K soils, single basal application showed no significant yield advantage, underscoring the importance of application timing. Split K application, particularly T4, also improved the grain quality traits, including protein content, wet gluten content, sedimentation value, and dough rheological properties. These improvements were associated with enhanced post-anthesis K uptake and KUE.

**Conclusion:**

Optimizing K application timing through split fertilization improves K utilization, yield, and grain quality. A site-specific strategy is recommended: 120kg K_2_O ha^-1^ applied in a 2:3:5 ratio (sowing/jointing/anthesis) for K-deficient eastern regions and K_2_O ha^-1^ with the same split ratio for high-K western regions. This approach provides a sustainable and efficient fertilization strategy for winter wheat production in the Huang-Huai-Hai region.

## 1 Introduction

Winter wheat (*Triticum aestivum* L.) is the most widely cultivated cereal crop and is fundamental to global food security (Mitura et al., 2023). Sustaining high-yield and grain quality under increased production demands requires effective nutrient management, particularly the optimized use of chemical fertilizers to enhance soil properties and support long-term agricultural sustainability (Panhwar et al., 2019). With the global population projected to reach 9.7 billion by 2050, food demand is expected to increase by more than 70%, leading to an increased reliance on chemical fertilizers (Lal, 2016). While nitrogen (N) and phosphorus (P) have historically dominated research and practice, the importance of potassium (K) in crop production has gained increasing recognition, as K can become a limiting factor for yield in many regions (Ali et al., 2019; Niu et al., 2013). In wheat, K plays multiple physiological roles, including enhancing photosynthesis, improving stress tolerance, and promoting overall crop growth (Sarwar et al., 2022; Rawat et al., 2022). Previous studies have demonstrated that judicious K application improves photosynthetic capacity, delays leaf senescence, and directly contributes to higher grain yields (Wang et al., 2020, Wang et al., 2025), underscoring its importance in modern wheat production systems.

Potassium (K) is fundamental to wheat physiology (White, 2000), playing a multifaceted role in maintaining cell function, regulating osmotic balance, activating enzymes, promoting photosynthesis, and assimilating transport (Ma et al., 2013; Hasanuzzaman et al., 2018; Sardans and Peñuelas, 2021). Collectively, these processes enhance stress tolerance, grain filling, and, ultimately, grain yield and quality. In conventional practice, K is typically applied as a single basal dose (Niu et al., 2013). However, K demand is relatively low during early vegetative growth and increases sharply from jointing to anthesis, a period during which approximately 50% of total lifetime K is accumulated (Ma et al., 2022). This dynamic requirement highlights a key limitation of the single basal application strategy, which often fails to sustain adequate soil-available K during peak demand (Cheng et al., 2024). Importantly, wheat K uptake is not constant but highly stage-specific (Zhan et al., 2016). Consequently, this temporal mismatch may result in insufficient K availability during critical grain-filling stages, leading to reduced fertilizer use efficiency and potential yield limitation. Staged (or split) K application has therefore been proposed as an effective strategy to better synchronize soil K supply with crop uptake patterns, thereby reducing fixation and leaching losses, improving nutrient use efficiency, and creating favorable physiological conditions for enhanced wheat productivity.

The efficacy of K management strategies is not universal but is strongly mediated by regional soil conditions (Shah and Wu, 2019). Substantial evidence has indicated that K uptake and crop responses vary markedly among different soil types and baseline fertility levels (Sattari et al., 2014; Wang et al., 2025; Zhan et al., 2016; Li et al., 2020; Niu et al., 2013). In major wheat-producing regions of China, such as the Huang-Huai-Hai Plain and North China Plain, significant east–west gradients exist in climate and soil properties, suggesting that optimal K management strategies must be region-specific (Han et al., 2025; Wang et al., 2025). Although previous studies have consistently demonstrated the yield advantages of split K application over a single basal dose, several important limitations are remaining. First, most studies have focused on individual sites, lacking systematic multi-regional comparisons. Second, research has primarily emphasized yield with limited evaluation of grain quality and economic returns. Third, split application strategies have typically focused on K distribution between sowing and jointing stages, whereas the potential benefits of anthesis-stage application—a critical period for grain filling and quality formation—remain insufficiently explored. Given the high K demand during this stage, associated with assimilate partitioning and grain development, incorporating anthesis-stage K application may further optimize nutrient supply and improve both yield formation and grain quality. Collectively, these gaps constrain the development of region-specific, high-efficiency K management strategies for diverse agro-environmental conditions.

Based on the stage-specific demand for K in winter wheat, we hypothesized that split K application, particularly with increased allocation during the jointing and anthesis stages, would better synchronize soil K supply with crop uptake compared with a single basal application. This improved synchronization is expected to enhance K accumulation, grain yield, grain quality parameters, and KUE across different soil K conditions. Furthermore, we hypothesized that the effectiveness of split K strategies would vary depending on the initial soil K status of different regions. To test these hypotheses, this study aimed to (1) evaluate the effects of two K application rates and four application methods—including a three-stage split application (sowing, jointing, and anthesis) on yield and grain quality of winter wheat across the contrasting eastern and western sub-regions of the Huang-Huai-Hai Plain, (2) assess the associated economic benefits, and (3) identify an optimal, region-specific K management strategy that synchronizes with crop demand across the growth cycle to improve KUE and promote sustainable, high-efficiency wheat production.

## 2 Materials and methods

### 2.1 Experimental sites and description

**Figure 1:**
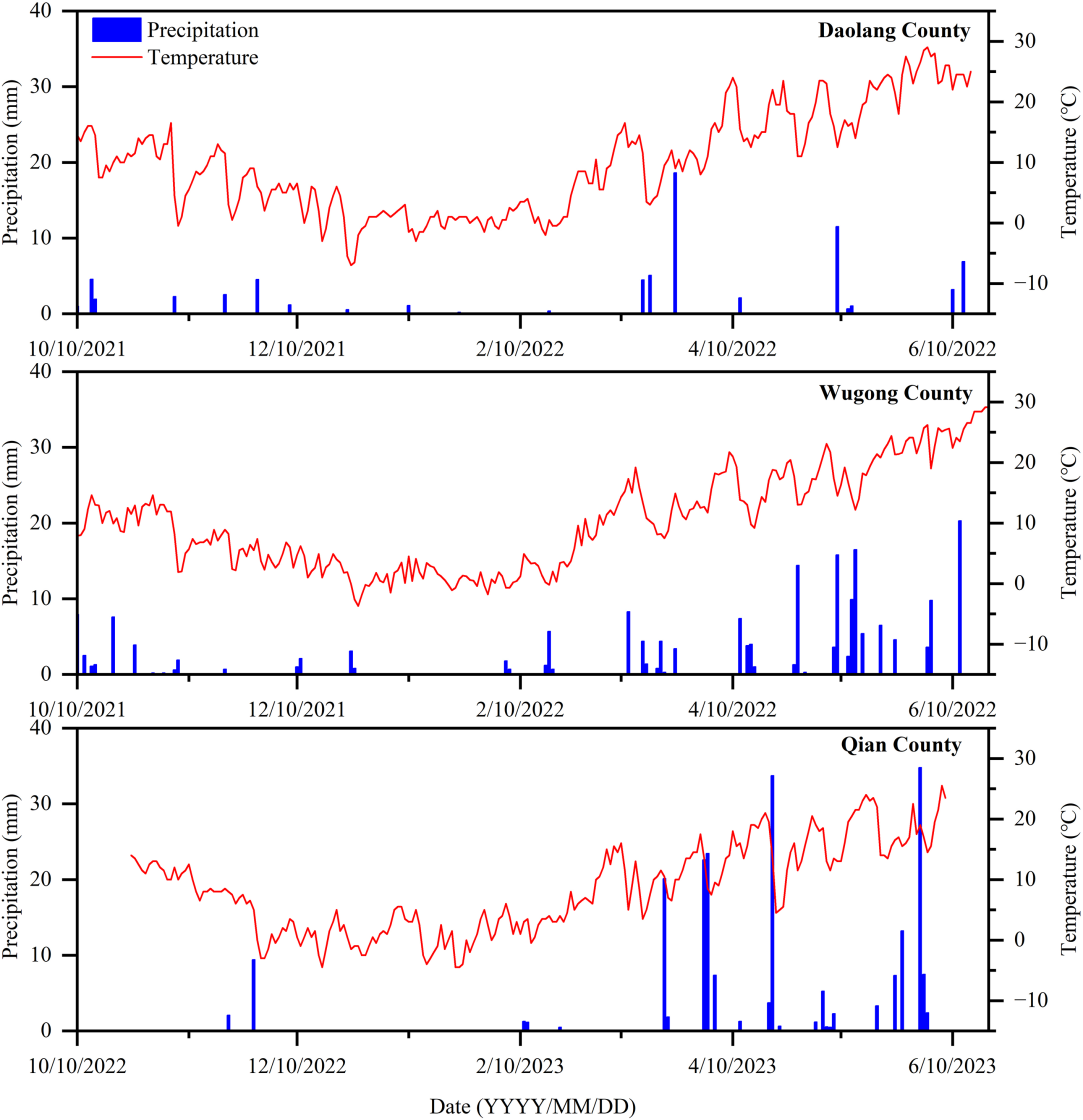
Daily average temperature and precipitation during the winter wheat growing seasons (2021–2023) at the experimental sites.

Field experiments were conducted during the 2021–2022 and 2022–2023 winter wheat growing season at three representative sites in China: Daolang Town, Tai’an City, Shandong Province (116°54′ E, 36°12′ N), Wugong Town, Wugong County, Xianyang City, Shaanxi Province (108°02′ E, 34°21′ N), and Chengguan Town, Qianxian County, Xianyang City, Shaanxi Province (108°24′ E, 34°53′ N). The altitudes of the experimental sites were 443, 350, and 568m, respectively. The regions are characterized by a warm temperate monsoon climate. The mean annual precipitation at the three sites was 654.6, 504.6, and 573mm, respectively, with corresponding mean annual temperature of 13.3 C, 14.4 C, and 12.6 C, respectively. The previous crop at all sites was maize, and the straw was crushed and incorporated into the soil after harvest. Daily temperature and precipitation during the experimental period are presented in **Figure 1**, and the physicochemical properties of the 0–20-cm soil layer before sowing are shown in **Table 1**.

**Table 1:** Basic information of experimental field.

Physicochemical properties	Units	Experimental sites		
		Daolang County	Wugong County	Qianxian County
Organic matter	(g·kg^-1^)	20.61	20.57	25.83
Total nitrogen	(g·kg^-1^)	1.22	1.27	0.8
Hydrolysable nitrogen	(mg·kg^-1^)	75.36	54.43	79.8
Available phosphorus	(mg·kg^-1^)	46.44	20.68	18.31
Available potassium	(mg·kg^-1^)	110.96	231.48	206.22
Soil classification		Brown earth soil	Lou soil	Lou soil

Brown earth soil corresponds to Udalfs (USDA Soil Taxonomy), and Lou soil corresponds to Anthrosols (WRB classification).

### 2.2 Experimental design and planting material

The experiment was conducted using a two-factor factorial randomized complete block design with three replicates. The two factors consisted of K application rate (K1: 120kg ha^-1^ and K2: 60kg ha^-1^) and K application method (T1–T5). The treatment with no K application (T1) served as the control. Four K application treatments were designed as a single application of K fertilizer applied as basal fertilizer before sowing (T2), two split applications of K fertilizer (50% applied as basal fertilizer before sowing and 50% applied as topdressing with irrigation water at the jointing stage (T3), three split applications of K fertilizer (20% applied as basal fertilizer before sowing, 30% applied as topdressing with irrigation water at the jointing stage, and 50% applied as topdressing with irrigation water at the anthesis stage (T4), and single application of controlled-release K fertilizer (all basal fertilizer applied) (T5). Each experimental plot measured 15m × 2m = 30 m^2^ and had three replicates. The strong-gluten wheat variety ‘Weilong 169’ was selected as the test material.

Potassium (containing 60% K_2_O) was used as the K fertilizer. Controlled-release K fertilizer was resin-coated (K content 44%, controlled-release period 90 days; supplied by Weituoer Company, Yangling District, Shaanxi Province). Triple superphosphate fertilizer (containing 46% P_2_O_5_) was used as the phosphate fertilizer, and urea (containing 46% N) was used as the nitrogen fertilizer. Moreover, 120kg ha^-1^ of P_2_O_5_ was applied as basal fertilizer before sowing, and 240kg ha^-1^ of pure nitrogen was applied, with 50% as basal fertilizer and the remaining 50% applied as topdressing with irrigation at the jointing stage. Furthermore, 120 or 60kg ha^-1^ of K_2_O was applied as topdressing according to the experimental design. In Daolang and Wugong, winter wheat for 2021–2022 was sown on October 18 and harvested on June 11, respectively. At Qianxian, winter wheat in 2022–2023 was sown on October 25 and harvested and measured on June 12.

### 2.3 Sampling and measurements

#### 2.3.1 Soil available potassium content

Soil samples were collected from the 0–60-cm soil profile at the jointing, anthesis, and maturity stages of winter wheat. The profile was divided into five depth intervals: 0–10, 10–20, 20–30, 30–40, and 40–60 cm. Within each experimental plot, three sampling points were randomly selected, and soil samples were collected separately from each depth layer at each point. For each depth interval, soil samples from the three sampling points were thoroughly mixed to form one composite sample per plot per layer. The composite samples were air-dried at room temperature, ground, and passed through a 1-mm sieve prior to analysis. Soil available K content was determined using 1mol L^-1^ NH_4_OAc and determined by flame photometry.

#### 2.3.2 Potassium content in plants

The dried plant samples were pulverized using a plant pulverizer, passed through a 100-mesh sieve, and digested with concentrated sulfuric acid. The total potassium content of each aboveground organ of the wheat plant was determined using H_2_SO_4_–H_2_O_2_ digestion and flame photometry.


$$\text{K accumulation in each organ }({\text{kg ha}}^{-1})=\text{Dry weight of each organ }\times \text{K content of each organ}$$

$$\text{K accumulation in above ground parts }({\text{kg ha}}^{-1})=\text{Sum of K accumulation in all organs at maturit}$$

$$\text{K allocation percentage in each organ }(\%)=\frac{\text{K accumulation in each organ}}{\text{K accumulation in aboveground parts}}\times 100$$

#### 2.3.3 Potassium utilization efficiency in winter wheat

Potassium fertilizer utilization efficiencies were calculated as follows:


$$\text{K fertilizer use efficiency }(\text{KUE},{\text{ kg kg }}^{-1})=\frac{\text{K accumulation in plants with K application}-\text{K accumulation in plants without K application}}{\text{K application rate }({\text{kg ha }}^{-1})}$$

$$\text{K fertilizer partial productivity }(\text{KPFP},{\text{ kg kg }}^{-1})=\frac{\text{Grain yield }({\text{kg ha }}^{-1})}{\text{K application rate }({\text{kg ha }}^{-1})}$$

$$\text{K fertilizer agronomic efficiency }(\text{KAE},{\text{ kg kg }}^{-1})=\frac{\text{Increased grain yield due to K application }({\text{kg ha }}^{-1})}{\text{K application rate }({\text{kg ha }}^{-1})}$$

#### 2.3.4 Grain yield and its components

During the winter wheat maturity period, 2.4 m² of wheat was harvested, threshed in each plot, and then air-dried. Subsequently, the moisture content was measured. The grains were weighed when the moisture content reached approximately 12.5%, and the yield was converted to hectare yield. The number of ears per unit area was investigated in 1 m² of each plot, and the effective number of ears per hectare was calculated. A total of 50 ears of wheat were randomly selected from each plot, and the number of grains per ear was investigated. From the grains harvested in each plot with a moisture content of 12.5%, 1, 000 grains were randomly selected and weighed to determine the thousand-grain weight. The 1000-grain weight was determined by weighing the grains. Each treatment was repeated three times.

#### 2.3.5 Economic benefit analysis

The grain income and economic benefits were calculated using the following formulas:


$$\text{Grain income }({\text{yuan ha}}^{-1})=\text{Grain yield }({\text{kg ha}}^{-1})\times \text{unit price }({\text{yuan kg}}^{-1})$$

$$\text{Economic benefits }({\text{yuan ha}}^{-1})=\text{Grain income }({\text{yuan ha}}^{-1})-\text{total cost }(\text{fertilizer cost}+\text{other cost})$$

#### 2.3.6 Determination of quality-related indicators for mature wheat grains

The protein content of whole wheat flour grains was determined using a semi-automatic Kjeldahl nitrogen analyzer (Kejltec™ 8100, FOSS Analytical A/S, Hillerød, Denmark), and protein content was calculated using a conversion factor of 5.7. Bulk density was measured using an HGT-1000 bulk density meter (Hangzhou Guangta Instrument Co., Ltd., Hangzhou, China). Wet gluten content was determined using a Perten 2200 gluten washing system (Glutomatic^®^ 2200, Perten Instruments AB, Hägersten, Sweden) according to GB/T14608-1993. The sedimentation value of the flour was determined using an Alpine sedimentation meter (Alpine, Augsburg, Germany). Dough rheological properties were evaluated using an Brabender extensograph (Extensograph-E, Brabender GmbH & Co. KG, Duisburg, Germany), following GB/T14615-2019. The farinograph parameters, including water absorption, dough development time, and stability time, were determined using a Brabender FarinoGraph (Brabender GmbH & Co. KG, Duisburg, Germany; supplied by Anton Paar Trading (Shanghai) Co., Ltd., Shanghai, China). Each sample was analyzed in triplicate, and mean values were used for statistical analysis.

### 2.4 Statistical analysis

Experimental data were processed using Microsoft Excel 2021 and analyzed using IBM SPSS Statistics 26.0 (SPSS Inc., Chicago, IL, USA). A two-factor analysis of variance (ANOVA) was conducted to evaluate the effects of potassium (K) application rate and application methods and their interaction. Prior to ANOVA, the data were checked for normality and homogeneity of variance, and the assumptions were satisfied. Mean comparisons were conducted using the least significant difference (LSD) test at *P*<0.05. A correlation analysis was performed to examine relationships among variables. Origin 2024b (OriginLab, Northampton, MA, USA) was used for data visualization.

## 3 Results

### 3.1 Dynamics of available potassium in different soil layers

**Figure 2:**
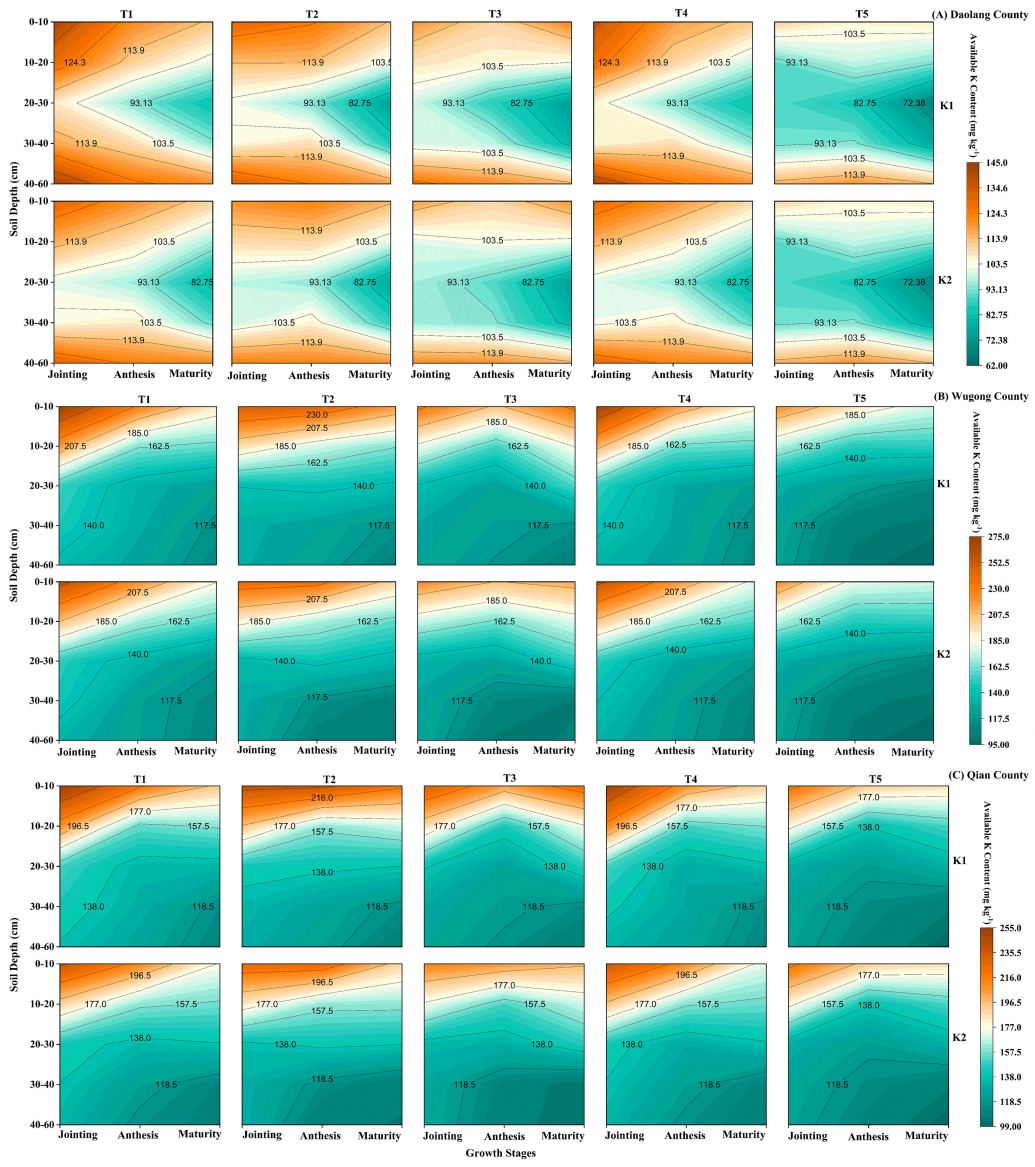
Dynamics of soil-available potassium within the 0–60-cm soil profile across key growth stages (jointing, anthesis, and maturity) under different potassium application treatments at three experimental sites, *viz*., **(A)** Daolang, **(B)** Wugong, and **(C)** Qian County.

Soil available K (AK) varied significantly among regions and treatments. The Wugong and Qianxian sites consistently showed higher AK (0–60 cm) than Daolang, and AK increased with K application rate (**Figure 2**), reflecting differences in baseline soil K status. At the jointing stage, both the single basal application (T2) and the three-split application (T4) significantly increased AK across the 0–60-cm soil profile. The higher AK under T2 indicates rapid K accumulation following basal application, whereas T4 maintained relatively high levels through split application, indicating an improved temporal distribution of K supply. By anthesis, the treatment effects diverged among regions. In surface soil (0–20 cm at Daolang and 0–30 cm at Wugong and Qianxian), split applications (T3 and T4) maintained higher AK than basal application, reflecting the effective replenishment of K during peak demand. In deeper layers, higher AK under T4 and T5 suggests a downward movement or redistribution of K. At maturity, T3 maintained higher AK in surface soil, highlighting the importance of late-stage K supply. In subsoil layers, relatively higher AK under T4 and T5 indicates continued redistribution or lower uptake at depth. Vertically, AK in the 0–60-cm profile declined over time at Wugong and Qianxian due to plant uptake. At Daolang, AK decreased in the 0–40-cm layer but increased in the 40–60-cm layer after jointing, indicating a downward movement under lower initial soil K conditions. Overall, single basal application increased the early-season K availability, particularly in deeper layers, whereas split applications improved the temporal and spatial distribution of K by maintaining higher availability in surface soil during the later growth stages.

### 3.2 Potassium accumulation, distribution, and translocation in winter wheat

**Figure 3:**
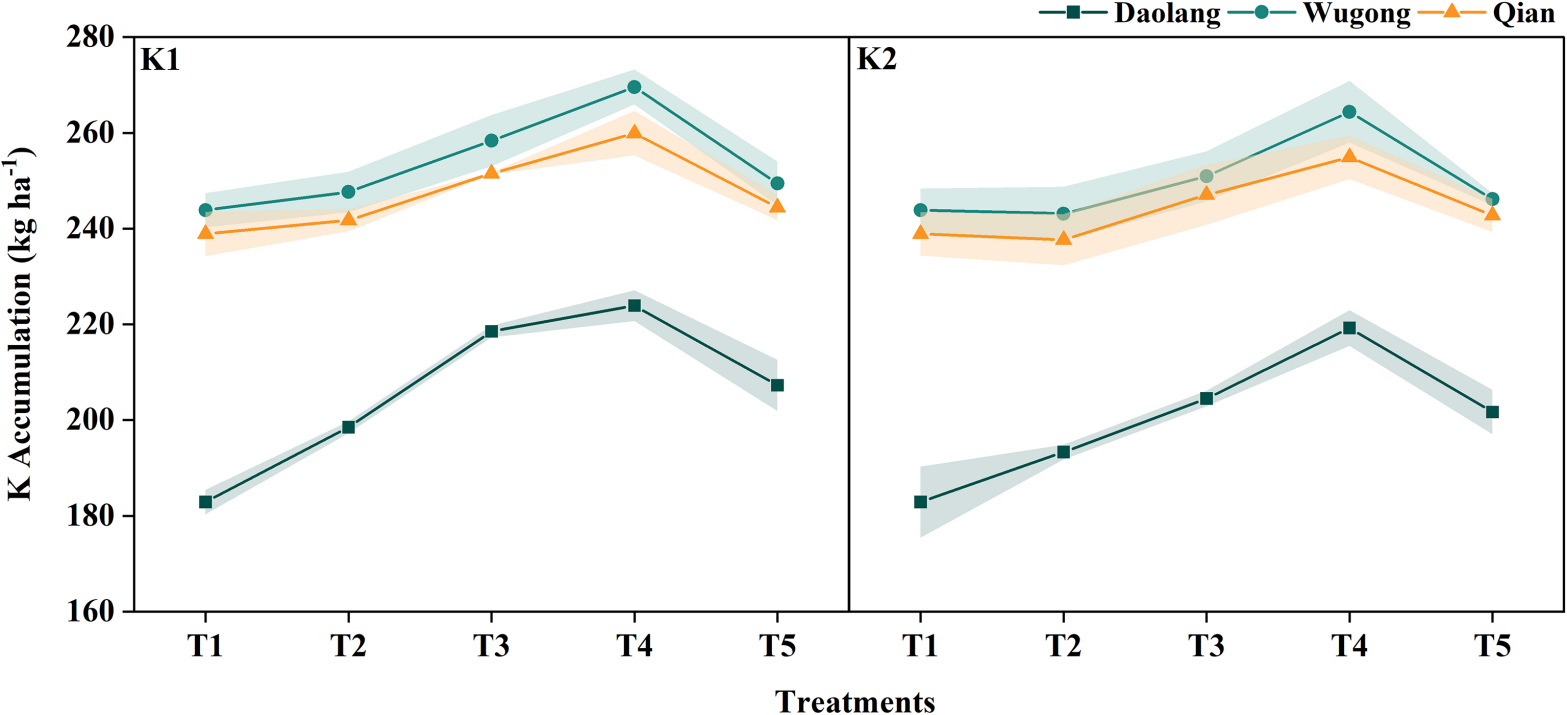
Potassium accumulation in winter wheat at the maturity stage under different potassium application treatments across three experimental sites (Daolang, Wugong, and Qian).

K accumulation in winter wheat at maturity increased with increasing K application rate. Across sites, K accumulation at maturity was consistently higher at the Wugong and Qianxian experimental sites than at the Daolang site (**Figure 3**). At all three sites, K accumulation was significantly lower under the lower K application rate of 60kg ha^-1^ compared with the higher rate (120kg ha^-1^). Under both K application rates, K accumulation at maturity followed the consistent order T4 > T3 > T5 > T2 > T1 across all experimental sites, with staged K application treatments exhibiting higher K accumulation than single-application and no-K treatments. At the Daolang site, all K fertilization treatments resulted in a significantly higher K accumulation at maturity than the no-K application (T1). Notably, at the high-K sites (Wugong and Qianxian), the single basal K applications (T2 and T5) failed to increase the total plant K accumulation compared to the control (T1), underscoring the necessity of split application in such environments.

**Figure 4:**
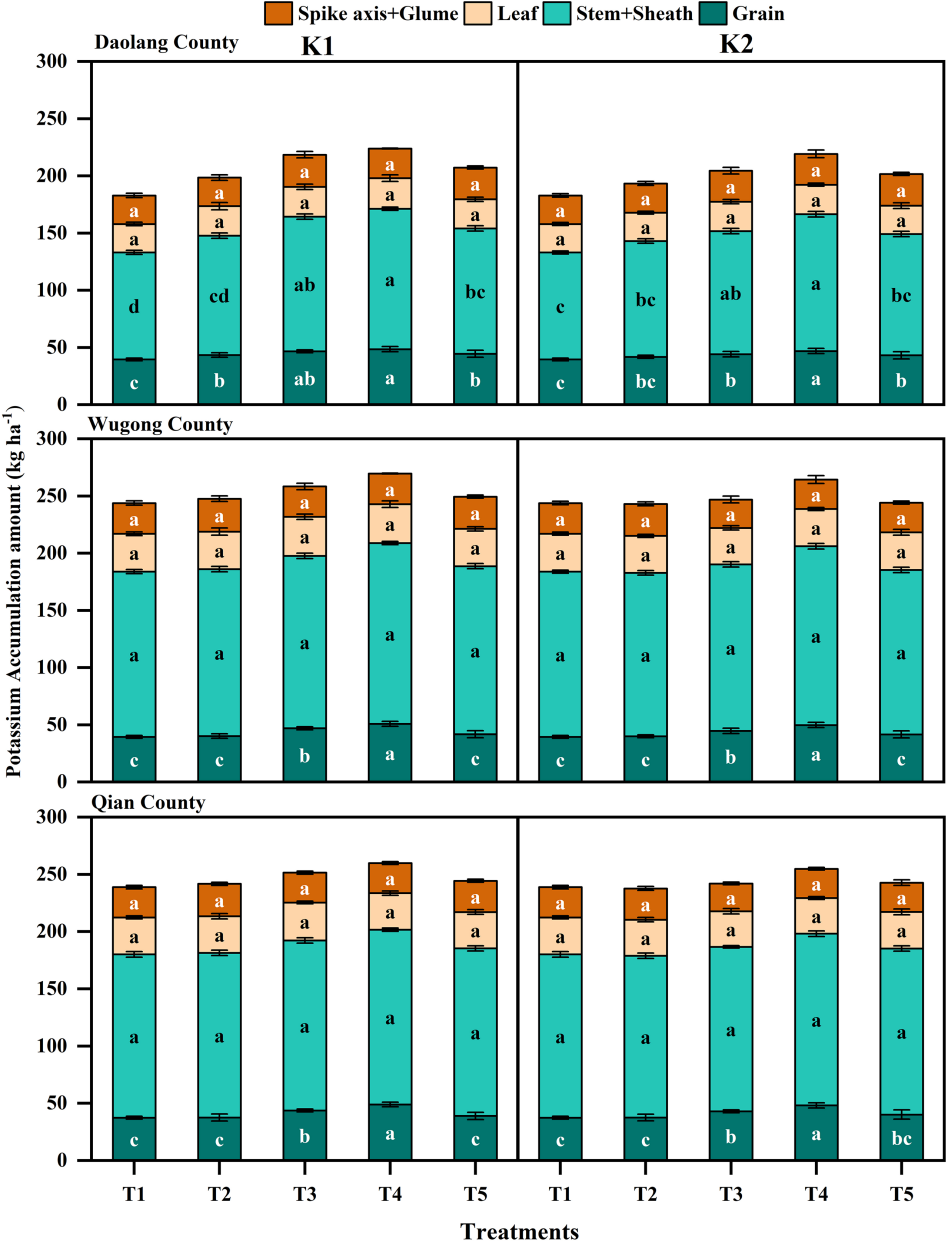
Potassium accumulation and distribution in different organs of winter wheat at the maturity stage under different potassium application treatments across three experimental sites: Daolang County, Wugong County, and Qian County. The vertical bars are means ± standard error, while lowercase letters above the bars indicate significant differences (*P*<0.05) between treatments.

Partitioning of K accumulation among plant organs further highlighted site-specific response (**Figure 4**). At Daolang site, treatment differences were primarily observed in stems and leaf sheaths and in grains, with K-applied treatments showing a significantly higher accumulation than the no-K treatment. Notably, K fertilization applied in three stages significantly exceeded other treatments. At Wugong and Qianxian sites, the treatment effects were mainly reflected in grain K accumulation, whereas K distribution in stems and leaf sheaths, leaves, and rachis and glumes showed relatively small differences among treatments.

Overall, K accumulation in winter wheat at maturity was positively associated with soil K supply, with higher K availability leading to greater plant K uptake. Staged K application was particularly effective in enhancing K accumulation, especially in low-soil-K environments, where significant increases in K accumulation were observed in both grains and stems and leaf sheaths compared with high-soil-K sites. These results indicate that regional soil K status should be considered when developing potassium management strategies.

### 3.3 Potassium distribution in different organs at maturity

**Figure 5:**
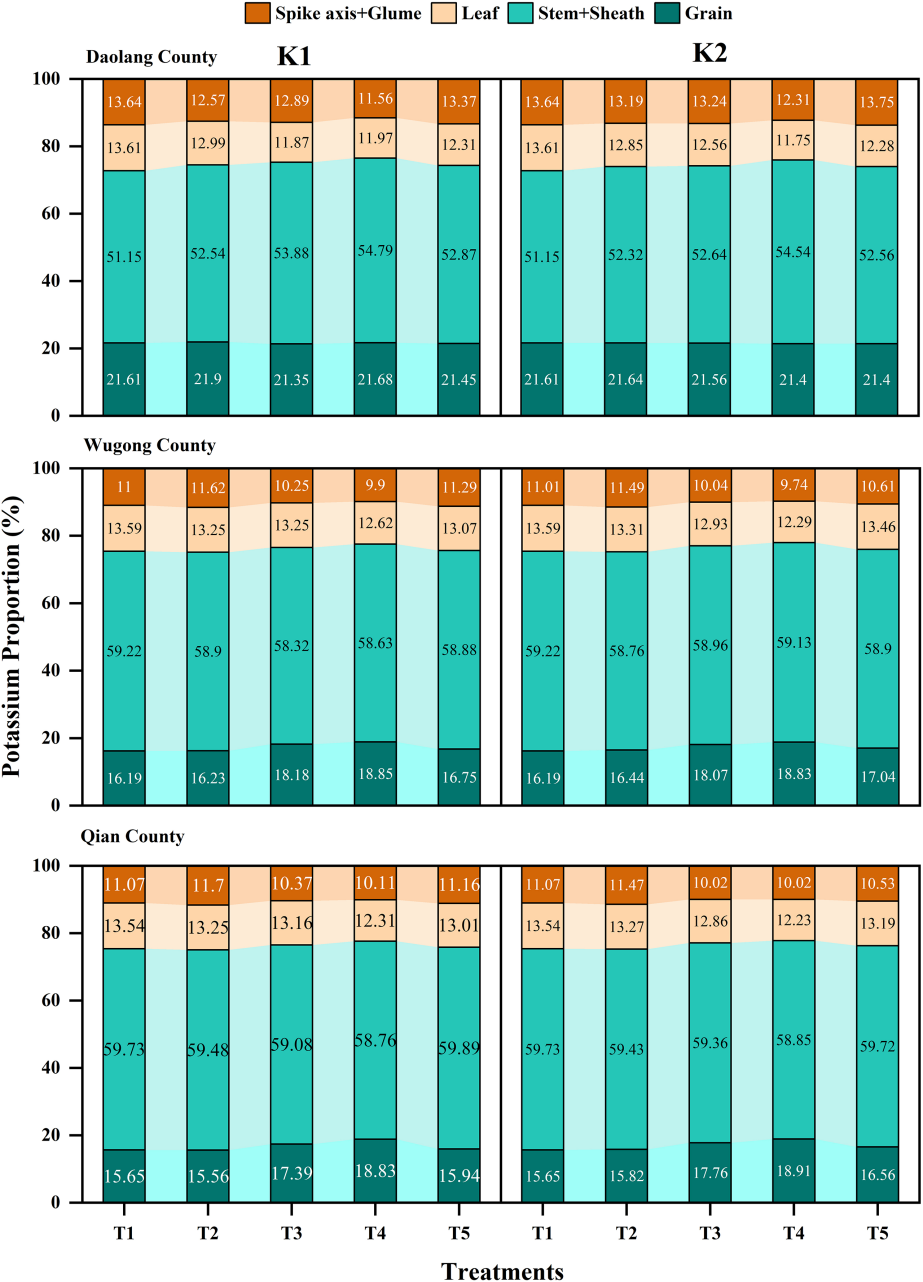
Proportional distribution of potassium accumulation among different organs of winter wheat at maturity stage under various potassium application treatments across three experimental sites: Daolang County, Wugong County, and Qian County.

The K distribution in different organs at maturity was generally consistent across all experimental sites (**Figure 5**). The maximum distribution was observed in the stem and leaf sheath, followed by the grain. Among different treatments, the order was T4 > T3 > T5 > T2 > T1. The distribution in leaves and rachis and glumes was relatively low. Within the same plot, different K application rates and application frequencies had little effect on the K distribution in different organs at maturity in winter wheat.

### 3.4 Winter wheat grain yield and potassium utilization efficiency

#### 3.4.1 Grain yield and yield components

**Figure 6:**
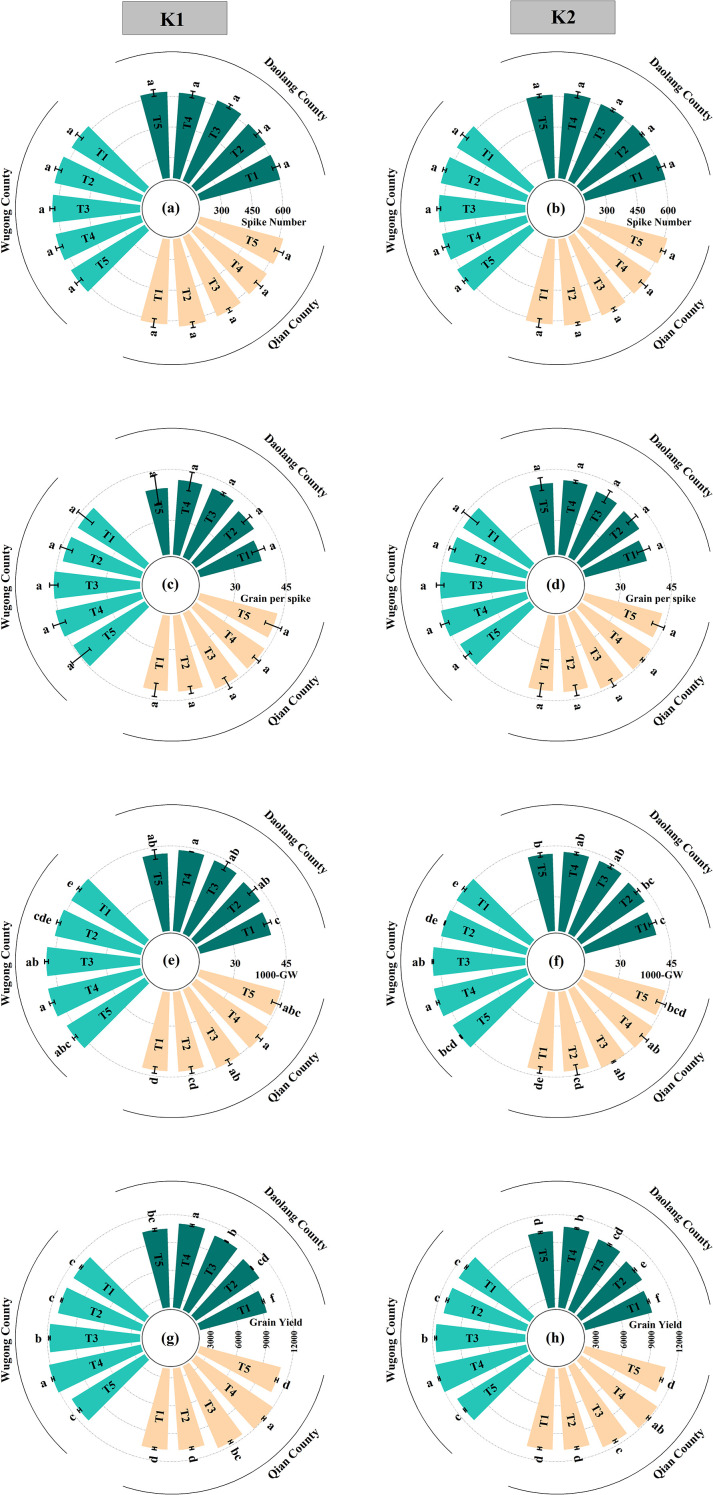
Grain yield and yield components of winter wheat under different potassium application treatments across three experimental sites (Daolang, Wugong, and Qian). The vertical bars are means ± standard error, while lowercase letters above the bars indicate significant differences (*P*<0.05) between treatments. **(a, b)** Spike number, **(c, d)** grains per spike, **(e, f)** thousand-grain weight (1, 000-GW), and **(g, h)** grain yield.

At the Daolang site, K application significantly increased the 1, 000-grain weight (TGW) compared with the no-K treatment (T1), with treatments ranked as T4 > T3 > T5 > T2 > T1. Grain yield showed a similar response pattern, with all K-applied treatments producing significantly higher yields than T1. Treatment T4 consistently achieved the highest yield at both application rates (K1 and K2), followed by T3 and T5, while no significant difference was observed between T2 and T1. Yield increases under K1 and K2 showed similar patterns, with split K application treatments exhibiting greater yield advantages than single basal application (**Figure 6**; **Supplementary Table 1**).

At the Wugong and Qianxian sites, yield responses to K management were primarily driven by changes in TGW. Grain yield followed the same order: T4 > T3 > T5 > T2 > T1. Treatments T3 and T4 significantly increased TGW relative to T2 and T1, whereas no significant difference was observed between T2 and T1. Under the same K application rate, T4 consistently produced significantly higher yields than the other treatments. The yield increases under K1 and K2 conditions were similar, indicating a limited response to increased K rate at these sites (**Figure 6**; **Supplementary Table 1**).

Across all three sites, split K application at the jointing and anthesis stages significantly increased the grain yield compared with 100% basal application at sowing. These results indicated that staged K application can effectively enhance wheat yield and compensate for reduced fertilizer inputs, particularly under conditions of moderate soil K availability (**Figure 6**; **Supplementary Table 1**).

#### 3.4.2 Correlation analysis between yield and its components

The relationship between yield and grain number per spike and TGW was consistent across three experimental sites, showing a significant positive correlation. However, the correlation between spike number and yield varied by location (**Table 2**). At Daolang site, grain number per spike showed a significant positive correlation with grain yield, while the spike number and grain yield at the Wugong and Qianxian sites showed no significant correlation. It should be noted that these correlations indicate statistical associations and do not imply causal relationships.

**Table 2:** Spearman correlation analysis between grain yield and its components at different sites.

Yield component	Grain yield		
	Daolang	Wugong	Qianxian
Spike number	0.44^*^	0.13^ns^	0.07^ns^
Grains per spike	0.63^**^	0.73^**^	0.62^**^
1, 000-grain weight	0.85^**^	0.89^**^	0.91^**^

ns, no significant correlation (p ≥ 0.05).

*p < 0.05 (significant); **p < 0.01 (significant).

#### 3.4.3 Yield increase at different locations

**Figure 7:**
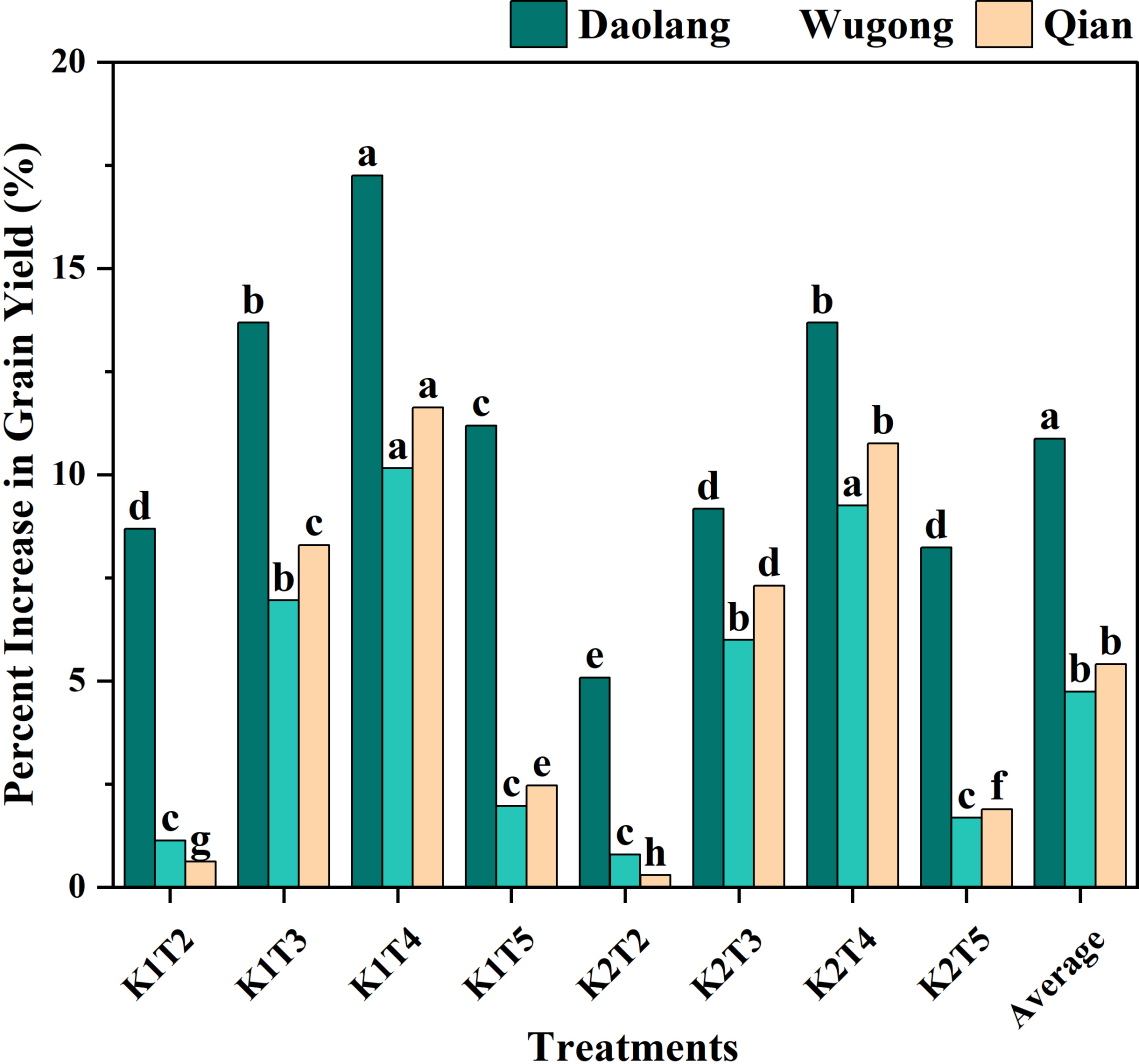
Percentage increase in grain yield of winter wheat under different potassium application treatments relative to the control (T1) across three experimental sites (Daolang, Wugong, and Qian). Different lowercase letters indicate significant differences among treatments within each site at *P* < 0.05 based on the least significant difference (LSD) test.

Yield increases generally followed the order K1T4 > K2T4 > K1T3 > K2T3 > K1T5 > K2T5 > K1T2 > K2T2, with the split K application treatments exhibiting a significantly higher yield increase than the other treatments (**Figure 7**). The average yield increase varied among experimental sites in the order Daolang > Qianxian > Wugong, with Daolang showing significantly higher increases than Wugong and Qianxian. At Daolang, all K treatments resulted in significant yield increases compared with the control. Staged K application produced greater increases than single-application basal K application, with treatment T4 significantly outperforming treatment T3. At Wugong and Qianxian, staged K application showed significant yield increases, while single basal K application had a very low yield increase. The results showed that staged K application had different effects in different regions. The yield increase was significant in areas with low soil K content, while the yield increase was smaller in areas with high soil K content.

#### 3.4.4 Potassium use efficiency

The K fertilizer use efficiency (KUE), agronomic efficiency (AE), and partial factor productivity (PFP) varied significantly with K application rate and timing across experimental sites (**Table 3**). At the K2 application rate, KUE, PFP, and AE were higher than at K1 across all treatments. Under the same K rate, split K applications (T3 and T4) consistently achieved significantly higher KUE, PFP, and AE compared with single basal applications (T2) at most sites. Correlation analysis revealed that KUE was significantly positively correlated with both PFP and AE at all sites and application rates, except at Qianxian under K2, where no significant correlation was detected between KUE and AE (**Table 4**). This indicates that improvements in PFP and AE collectively enhanced KUE in winter wheat.

**Table 3:** Effects of different treatments on potassium fertilizer utilization rate, partial productivity of K fertilizer, and agronomic efficiency of potassium fertilizer.

Site	Application method	KUE	PFPk	AEk
		(kg kg^-1^)	(kg kg^-1^)	(kg kg^-1^)
Daolang	T1	–	–	–
	K1T2	13.54b	85.42f	6.82e
	K1T3	29.88ab	89.34de	10.75cd
	K1T4	30.45ab	92.16d	13.56b
	K1T5	18.39b	87.40ef	8.80de
	K2T2	18.28b	165.17c	7.98e
	K2T3	36.73ab	171.60b	14.41b
	K2T4	54.04a	178.69a	21.50a
	K2T5	27.69b	170.09b	12.90bc
Wugong	T1	–	–	–
	K1T2	3.56b	92.78d	1.03d
	K1T3	12.13ab	98.12d	6.38c
	K1T4	17.63ab	101.05d	9.31b
	K1T5	2.58b	93.56d	1.81d
	K2T2	1.6241b	184.94c	1.46d
	K2T3	5.51ab	194.48b	11.00b
	K2T4	27.02a	200.45a	16.96a
	K2T5	1.84b	186.54c	3.06d
	T1	–	–	–
Qianxian	K1T2	2.78a	89.95e	0.55g
	K1T3	10.56a	96.80d	7.41d
	K1T4	13.78a	99.79d	10.40c
	K1T5	2.57a	91.59e	2.20f
	K2T2	1.37a	179.31c	0.51g
	K2T3	5.70a	191.86b	13.07b
	K2T4	19.47a	198.04a	19.24a
	K2T5	2.21a	182.17c	3.38e
F-Value	K method (T)	28, 00***	45518.4***	636.04***
	Site (S)	40.31***	692.51***	180.12***
	K rate (K)	1.53ns	77333.60***	208.91***
	T × S	3.12**	50.10***	21.32***
	T × K	2.32ns	4857.73***	49.03***
	S × K	3.57*	140.82***	0.62ns
	T × S × K	0.33ns	9.79***	1.49ns

ns, no significant correlation (p ≥ 0.05).

*p Values followed by different lowercase letters indicate significance at *p < 0.05 (significant); **p < 0.01 (significant); ***p < 0.001 (significant).

**Table 4:** Correlation analysis among KUE parameters at different sites.

K application rate (kg ha^-1^)		KUE (kg kg^-1^)		
		Daolang	Wugong	Qianxian
K1	PFPk	0.77^**^	0.81^**^	0.77^**^
	AEk	0.66^*^	0.71^**^	0.64^*^
K2	PFPk	0.83**	0.77**	0.67*
	AEk	0.64*	0.645*	0.51 ns

ns, no significant correlation (p ≥ 0.05).

*p<0.05 (significant); **p<0.01 (significant). PFP, Partial factor productivity; AE, Agronomic efficiency of K fertilizer.

Potassium application method significantly influenced KUE, with the ranking T3 > T4 > T2 across sites. Site-specific differences followed the order Daolang > Wugong > Qianxian, with K2T3 at Daolang achieving the highest efficiency and K2T1 at Qianxian the lowest. Partial factor productivity differed significantly between K rates due to application amount, with K2 treatments consistently higher than K1. Within each K rate, PFP and AE followed the same ranking: T3 > T4 > T2, with T3 significantly outperforming other treatments. Increasing the K application rate enhanced the wheat yield but reduced the KUE, PFP, and AE. Split K applications improved all efficiency parameters across sites, although the magnitude of improvement varied by location, being greatest at sites with lower initial soil K content (Daolang) compared with higher-K sites (Wugong and Qianxian).

### 3.5 Net economic benefit

**Figure 8:**
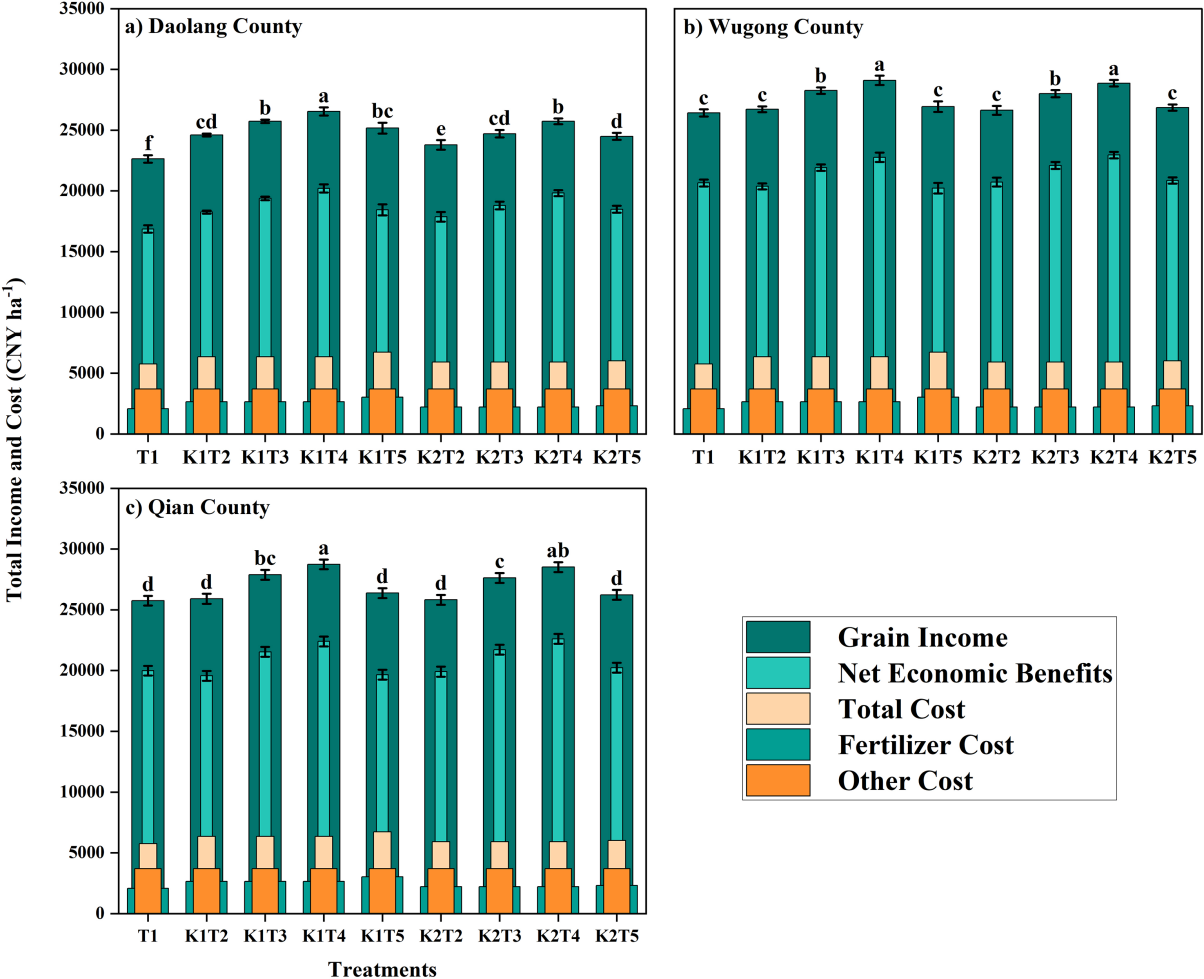
Grain income, total cost, and net economic benefits of winter wheat under different potassium application treatments across three experimental sites: **(a)** Daolang County, **(b)** Wugong County, and **(c)** Qian County. Economic components include grain income, net economic benefits, total cost, fertilizer cost, and other costs (CNY ha^-1^). The vertical bars are means ± standard error, while lowercase letters above the bars indicate significant differences (*P* < 0.05).

Split K application treatments resulted in significantly higher net economic benefits (NEB) than other treatments across all experimental sites, although the magnitude of improvement varied by location (**Figure 8**; **Supplementary Table 2**). At the Daolang site, both grain revenue and NEB under K fertilization was significantly higher than the no-K control, with NEB generally increasing with K application rate. Among treatments, K1T3 achieved the highest NEB, although it was not significantly different from K2T3. At the Wugong and Qianxian sites, split K applications consistently outperformed single basal applications at equivalent K rates. Although higher K rates (K1) increased the gross revenue, NEB was greater under the lower K rate (K2) due to reduced input costs, with K2T3 yielding the highest returns at both sites.

The economic efficiency of K fertilization was strongly site-dependent and closely related to soil K status. At the low-K Daolang site, K application substantially increased both gross revenue and NEB, with greater gains observed at higher K rates. In contrast, at the Wugong and Qianxian sites, increasing K rates enhanced the yield but reduced the net returns, indicating diminishing economic efficiency. Notably, single basal K applications reduced the NEB at these sites, whereas split applications significantly improved profitability. Within the tested K rates, application timing had a greater influence on economic returns than total K input, particularly under moderate to high soil K conditions. These findings highlight that economically optimal K management should be site-specific, with split K application providing a more efficient and profitable strategy across diverse soil K conditions.

### 3.6 Effects of split potassium application on winter wheat grain quality

#### 3.6.1 Grain quality parameters

Split K application significantly affected the grain quality parameters; however, the magnitude and direction of the response varied among traits and experimental sites (**Table 5**). In general, treatments involving split K application (T3 and T4) improved the protein content, wet gluten content, and sedimentation value compared with the control (T1) and single basal application (T2). The test weight showed no consistent response to K management. The Daolang site exhibited higher protein, wet gluten, and sedimentation values than Wugong and Qianxian, reflecting differences in soil K status. Across sites, both T3 and T4 treatments enhanced the grain quality; however, T4 frequently exhibited the highest values for key grain quality parameters, followed by T3, while T2 showed comparatively limited improvements. At Daolang (low soil K), split K application markedly improved all quality parameters. At Wugong and Qianxian (higher soil K), both T3 and T4 improved the grain quality relative to T1 and T2, although differences between these two split treatments were trait dependent.

**Table 5:** Changes of grain quality indexes of winter wheat under different treatments.

Experimental site	Potassium application method	Winter wheat grain quality				Dough rheological properties				Farinographic indexes		
		Protein (%)	Bulk density (g L^-1^)	Wet gluten (%)	Sedimentation (mL)	Stretch area (cm²)	Stretch resistance (E.U.)	Extensibility (mm)	Maximum resistance to stretch (E.U.)	Absorption (%)	Development (min)	Stability (min)
Daolang	T1	13.05c	834.77a	30.51c	31.87c	85.59b	315.38a	146.24a	475.86bc	60.08a	3.94c	13.62d
	K1T2	13.17bc	828.33a	31.30bc	33.44ab	96.91ab	319.49a	148.8a	485.48bc	59.92a	4.10c	15.87d
	K1T3	13.25ab	835.48a	32.11ab	33.90ab	95.37ab	327.51a	142.59a	533.32ab	59.60a	4.47ab	16.85ab
	K1T4	13.42a	834.96a	33.47a	33.97a	106.73a	334.53a	143.88a	551.27a	59.96a	4.64a	17.03a
	K1T5	13.12bc	830.64a	32.06bc	33.76ab	100.42ab	318.64a	145.56a	486.52bc	59.67a	4.35b	16.18a
	K2T2	13.09bc	833.42a	31.77bc	32.54bc	93.95ab	318.03a	144.46a	480.35bc	59.27a	4.08c	13.78b
	K2T3	13.18bc	840.08a	32.51ab	32.88bc	100.09ab	326.49a	147.63a	526.73ab	60.43a	4.43b	15.59c
	K2T4	13.29ab	832.38a	32.63ab	33.64ab	105.38a	332.36a	140.05a	548.96a	60.26a	4.65a	16.40abc
	K2T5	13.08bc	831.10a	31.92bc	32.80bc	94.22ab	320.35a	143.90a	473.63c	59.88a	4.35b	14.07d
Wugong	T1	12.20d	819.91a	29.27e	31.24b	80.19c	306.29a	148.34a	442.30c	59.51a	3.90d	13.09d
	K1T2	12.26d	817.67a	31.37bcd	31.76ab	84.27bc	308.88a	149.10a	449.35c	60.12a	3.92d	13.09d
	K1T3	13.08ab	811.42a	32.43ab	32.84ab	95.67ab	319.81a	151.91a	499.51ab	59.51a	4.19bc	15.75b
	K1T4	13.22a	815.62a	33.61a	34.16a	100.59a	326.45a	150.64a	525.66a	60.41a	4.41a	17.03a
	K1T5	12.49c	811.33a	31.55bc	32.55ab	90.68bc	314.38a	147.16a	466.94bc	60.09a	3.97d	14.63c
	K2T2	12.24d	825.09a	29.34e	32.27ab	82.33bc	308.75a	141.62a	448.60c	60.32a	3.92d	13.09d
	K2T3	12.95b	820.42a	29.66cde	32.61ab	95.83ab	317.39a	149.13a	484.75ab	60.39a	4.03cd	15.67b
	K2T4	13.12ab	813.24a	29.85cde	33.44ab	98.04ab	322.43a	151.35a	514.74a	60.68a	4.29ab	16.88a
	K2T5	12.34cd	813.81a	29.46de	32.76ab	88.06bc	311.22a	144.32a	462.08bc	58.70a	3.96d	13.74cd
Qianxian	T1	11.96f	817.67a	29.40d	29.32c	86.27b	312.25a	155.43a	456.21d	59.73a	4.06c	14.93def
	K1T2	12.11def	819.38a	30.40cd	33.19ab	93.12ab	314.92a	151.47a	460.42d	60.34a	4.09c	15.08def
	K1T3	12.70b	814.06a	32.50abc	33.56a	95.50ab	324.19a	148.75a	523.54ab	59.49a	4.34ab	16.23ab
	K1T4	12.92a	812.80a	33.74a	33.89a	102.50a	330.66a	150.07a	534.10a	59.58a	4.51a	16.94a
	K1T5	12.30cd	814.94a	30.88bcd	33.40a	96.90ab	316.26a	149.39a	480.55bcd	59.40a	4.19bc	15.94abc
	K2T2	12.03ef	818.93a	29.67d	30.76bc	90.69ab	312.27a	144.93a	459.87d	59.70a	4.07c	14.53ef
	K2T3	12.47c	824.43a	31.49abc	32.81ab	94.36ab	319.75a	145.01a	490.15bcd	58.95a	4.35ab	15.61bcd
	K2T4	12.87ab	818.50a	32.88ab	32.87ab	99.97ab	326.34a	146.69a	521.14abc	58.34a	4.50a	16.68a
	K2T5	12.21de	819.99a	30.49cd	32.28ab	95.48ab	317.69a	145.04a	478.30cd	59.20a	4.13c	14.77ef
*F*-value	K method (T)	3, 918.93a	1.69ns	122.95a	16.24a	121.17a	31.23a	10.47a	391.45a	3.38*	45, 501.2a	122.42a
	Site (S)	11, 536.7a	35.27a	68.95a	4.99b	53.13a	19.28a	36.48a	146.10a	13.96a	26, 775.7a	33.48a
	K rate (K)	290.87a	3.47ns	101.45a	5.86b	6.01b	1.77ns	30.09a	24.41a	2.42ns	976.49a	43.83a
	T × S	449.47a	1.51ns	6.10a	1.45ns	4.86a	0.14ns	11.28a	3.77c	6.91a	1, 958.92a	7.71a
	T × K	26.82a	1.09ns	10.43a	0.42ns	2.41ns	0.38ns	6.44a	4.81c	3.22b	161.13a	5.69a
	S × K	2.95ns	0.23ns	48.10a	1.49ns	0.03ns	0.17ns	3.21b	0.88ns	5.65c	274.68a	8.91a
	T × S × K	9.18a	0.51ns	4.25a	0.47ns	0.89ns	0.13ns	2.28b	1.83ns	4.08a	223.98a	1.18ns

Effects of different treatments on grain tensile area, tensile resistance, extensibility, and maximum elongation resistance; farinographic indexes of winter wheat under different treatments.

*F*-value indicates analysis of variance. Values followed by different lowercase letters indicate at difference significance levels.

ns, non-significant differences.

#### 3.6.2 Dough rheological properties

Extensograph parameters—extensibility area, resistance at 50-mm extension, and maximum resistance—followed the ranking T3 > T2 > T4 > T1 across sites (**Table 5**). The maximum resistance in T3 was significantly higher than in T4, T2, and T1. Extensibility remained unchanged across treatments. Split K applications enhanced the dough strength characteristics, with T3 demonstrating the most pronounced improvements in resistance properties, indicating enhanced gluten network development.

#### 3.6.3 Farinograph quality parameters

Dough development time and stability time were significantly higher in T2 and T3 than in the other treatments, following the order T3 > T2 > T4 > T1 (**Table 5**). Water absorption showed no significant response to K treatments across sites. At Daolang, all K treatments increased the dough development time and stability compared with the control, with split applications (T3, T4) outperforming single basal application (T2). At Wugong and Qianxian, split K applications substantially improved the farinograph parameters, whereas basal-only application (T2) had minimal effects. Treatment T3 consistently produced dough with superior mixing tolerance and stability across all soil K conditions. Split K application, particularly the 2:3:5 timing ratio (T3), enhanced the wheat processing quality by improving protein accumulation and gluten strength development. The magnitude of quality improvements was greater at low soil K sites, though split applications provided quality benefits across all K availability levels.

## 4 Discussion

### 4.1 Impact of K fertilizer application on soil-available potassium dynamics

Potassium (K) plays a critical role in sustaining wheat productivity, yet its availability is highly dynamic and often poorly synchronized with crop demand under conventional fertilization (Römheld and Kirkby, 2010; Mosaad and Fouda, 2016). Maintaining adequate soil-available K is essential for crop uptake, particularly under intensive N and P fertilization regimes that may exacerbate K deficiency. Soil K exists in three interconvertible pools: available, slowly available, and fixed—whose distribution is governed by soil physiochemical properties (Bell et al., 2020). Although these pools remain in dynamic equilibrium, fertilizer management can disrupt this balance and influence K availability and plant uptake (Das et al., 2022). Conventional single basal application at sowing is inefficient, as excess K supplied during early vegetative growth is susceptible to leaching due to its mobility in soil (Wang et al., 2025; Singh et al., 2000). Consequently, K availability declines during the critical jointing-to-anthesis period, leading to reduced KUE and crop performance. In contrast, split K application has been proposed to better synchronize nutrient supply with crop demand and improve KUE.

The present multi-site study across the Huang-Huai-Hai region provides evidence supporting the superiority of split over single basal K application. Consistent with previous findings (Wang et al., 2025; Vijayakumar et al., 2024; Lu et al., 2014 and Niu et al., 2013), split K application sustained higher soil-available K (AK) throughout the crop growth cycle. Specifically, single basal application increased the AK content in the 0–60-cm soil profile at jointing, but this effect declined toward anthesis due to K redistribution and depletion. In contrast, split treatments (T3 and T4) maintained higher AK levels during later growth stages, particularly in surface soil layers, indicating improved synchronization between K supply and crop demand.

Site-specific responses to split K application were also evident. At the low-K Daolang site, split application increased the AK content in the 0–20-cm layer during anthesis and maturity, whereas at Wugong and Qianxian, this benefit extended to the 0–30-cm layer (**Figure 2**). Vertical distribution patterns differed among sites, likely reflecting variations in soil type, K buffering capacity, and transformation among K pools. The three-split strategy (2:3:5 at sowing, jointing, and anthesis) was more effective than the two-split strategy (5:5 at sowing and jointing) in maintaining soil AK after jointing, consistent with peak crop K demand during this period (Li et al., 2017).

In contrast, the controlled-release K treatment (T5) did not perform as effectively as the split applications in maintaining soil K availability during the critical growth stages. Although T5 increased the early-season K supply, its release pattern is governed primarily by environmental conditions rather than crop demand, which may lead to insufficient K availability during the jointing–anthesis period. In addition, the basal placement of controlled-release K may result in partial redistribution or fixation before peak uptake, reducing its effectiveness in the root zone. These limitations explain why T5 underperformed relative to split applications, particularly at sites with higher native soil K where the precise timing of nutrient supply is more critical than sustained release.

Overall, these results demonstrate that split K application improves the temporal distribution of soil-available K and enhances its synchronization with crop demand. This improved alignment supports plant K uptake, photosynthesis, assimilate transport, and reproductive development, ultimately contributing to higher yield and improved grain quality. The findings highlight the importance of optimizing K application timing to maximize productivity and nutrient use efficiency under varying agro-environmental conditions.

### 4.2 Impact of K fertilizer application on grain yield and its components of winter wheat

K fertilization mitigates soil K depletion, replenishes the available soil K pool, and improves crop yield, with reported increases ranging from 5% to 19% depending on soil fertility conditions (Wang et al., 2024; Li et al., 2017). However, the magnitude and direction of yield response are strongly dependent on initial soil K status. In K-deficient soils, such as those in the eastern Huang-Huai-Hai region, K application significantly enhances yield formation, whereas responses are comparatively limited or even neutral in the K-rich soils of the western Huang-Huai-Hai region (Peng et al., 2016). In some high-K soils, excessive basal K application may even reduce grain yield (Dhillon et al., 2019). Split K application can partly overcome this limitation by synchronizing K supply with crop demand, and previous work has shown that interactions between application frequency and total K rate can enhance the yield more effectively than single application (Wang et al., 2025; Lu et al., 2014). Nevertheless, the reported effects of split K application on yield components—including spike number, grains per spike, and thousand-grain weight (TGW)—have been inconsistent, which is likely because of differences in soil fertility conditions and wheat varieties (Wu et al., 2022). These associations suggest potential relationships; however, they do not establish direct causal effects.

The present study provides a consistent and spatially robust dataset from three sites representing contrasting K fertility regimes and climatic conditions. Across all sites, split K application increased the grain yield primarily by improving grains per spike and TGW, with TGW contributing more strongly to final yield. This finding aligns with the findings of Wang et al. (2025) but contrasts with those of Adnan et al. (2016). The superior yield under the three-stage split application can be attributed to improved synchronization between K supply and crop demand during critical growth stages, particularly from jointing to anthesis. This pattern is physiologically plausible because the jointing-to-anthesis period coincides with stamen and pistil primordia differentiation, during which sufficient K supply is critical for floret fertility and grain set (Ma et al., 2019). The timely K application during this window likely supports the formation and survival of more fertile florets, thereby increasing the grains per spike. Furthermore, adequate K availability during the post-anthesis period delays leaf senescence, sustains photosynthetic activity, and promotes assimilate partitioning to the grain, thereby increasing the 1, 000-grain weight (Wang et al., 2025). These effects are mediated through K-regulated stomatal conductance, enzyme activation, and osmotic adjustment, which collectively maintain carbon assimilation during grain filling (Ahmad et al., 2018; Prajapati et al., 2026).

Between-site differences in yield components further support the importance of both soil K status and climate. The number of grains per spike at Wugong and Qianxian was higher than at Daolang, which may reflect the higher native soil K levels at Wugong and Qianxian, which provide adequate K supply during early reproductive development for spike formation. This result is consistent with that of Zhao et al. (2014). Similarly, TGW at Wugong and Qianxian exceeded that at Daolang, which is likely because the local climatic conditions during grain filling at these sites were more favorable for assimilate accumulation and kernel development. These findings indicate that the site-specific soil and climatic conditions modulate how K management translates into changes in yield components.

The stronger yield response at the low-K Daolang site reflects the greater limitation of K under deficient conditions, which enhances the effectiveness of fertilization. This confirms the findings of Sarwar et al. (2022) which reported that wheat yield improved under K application. In contrast, at the higher-K Wugong and Qianxian sites, single basal K application had no significant yield-increasing effect compared with control, whereas treatments involving topdressing at jointing and anthesis stages showed significant yield increases. These results are consistent with those of Sharma et al. (2022) and support the concept that, in high-fertility soils, application timing matters more than the total rate. These differences suggest that, in low-K soils, both split K applications are effective for increasing yield, while in soils with higher native K, the timing and distribution of K topdressing become more important than a simple increase in total applied K.

Taken together, the multi-site results support a differentiated K management strategy for winter wheat. In regions with low soil K content, K fertilization substantially increases yield: single basal application improves yield compared with no K, and split K application further enhances yield and KUE. Therefore, under such conditions, K should be applied at sowing and complemented with topdressing at later stages. In regions with high soil K content, single basal application of K fertilizer had no significant effect on increasing the wheat yield, and management should emphasize topdressing during the later growth stages. Moreover, splitting K between jointing and anthesis is more effective than applying it only at jointing, as this approach better matches crop demand during the critical periods of floret fertility and grain filling (Wang et al., 2025). These insights provide a practical basis for optimizing K fertilization regimes according to local soil K status and climatic conditions in winter wheat production systems.

### 4.3 Effects of K fertilizer strategy on KUE in winter wheat

The KUE is strongly influenced by both application rate and timing, reflecting the extent to which applied nutrients are effectively taken up and utilized by the crop. In the present study, split K application enhanced KUE, AE, and PFP, indicating improved nutrient utilization under optimization management. These findings are consistent with previous studies demonstrating that appropriate K fertilization strategies can significantly improve fertilizer use efficiency in cereal systems (Khatun et al., 2022; Vijayakumar et al., 2024). The improved efficiency under split application can be attributed to better synchronization between soil K availability and crop demand. By supplying K at critical growth stages, particularly jointing and anthesis, nutrient availability is maintained during peak uptake periods, supporting physiological processes such as assimilate transport and grain yield formation (Ishfaq et al., 2023). This temporal alignment enhances plant K uptake and reduces inefficiencies associated with early-season nutrient surplus.

Application rate also influenced efficiency, with higher KUE, AE, and PFP observed at the lower K rate (K2), reflecting the typical inverse relationship between application rate and efficiency metrics. Split K applications consistently improved KUE, PFP, and AE compared with single basal application, highlighting the importance of synchronizing the nutrient supply with crop uptake dynamics. This pattern is consistent with those found by Saudy et al. (2023) and Wang et al. (2025). Correlation analysis further confirmed strong positive relationships between KUE, PFP, and AE across most sites, indicating that improved nutrient uptake is closely linked with enhanced productivity. The lack of correlation at Qianxian under K2 suggests site-specific constraints affecting nutrient utilization. Site-specific differences further highlight the role of soil fertility. Greater efficiency gains were observed at the low-K Daolang site, whereas responses were smaller at Wugong and Qianxian sites with higher baseline soil K. This indicates that split application provides larger benefits under K-limited conditions. This finding is supported by Xu et al. (2025), who showed greater KUE response to split K in low-fertility soils.

From an environmental perspective, improved KUE under split application may reduce potential nutrient losses by minimizing excess soil K and improving nutrient retention. However, these losses were not directly quantified in this study, and future research should include environmental assessments. Additionally, while KUE indices capture agronomic responses, more comprehensive approaches such as nutrient balance analysis would further improve the evaluation of KUE. From an economic and practical standpoint, split K application can be effectively implemented through fertigation in irrigated systems, reducing labor constraints while improving nutrient delivery. However, its adoption should consider local management conditions, particularly in rainfed or labor-limited systems. Overall, split K application represents a practical and efficient strategy to improve KUE in winter wheat.

### 4.4 Effects of potassium fertilization on grain quality of winter wheat

Grain quality in winter wheat is primarily determined by genotype, environment, and their interaction, with genotype providing the genetic basis and environmental and management conditions modulating trait expression. Traits such as grain hardness, water absorption, and test weight are predominantly genotype-driven and show limited response to agronomic management (Johansson et al., 2020). Consistent with this, K application methods had no significant effects on test weight or water absorption across sites in the present study. In contrast, parameters such as protein content, wet gluten content, starch composition, and sedimentation value are more sensitive to environmental factors and nutrient management (Gu et al., 2021).

Previous research indicated that K fertilization can improve wheat processing quality by increasing protein content, wet gluten content, sedimentation value, and gluten polymerization index (insoluble gluten/total gluten) as well as improving dough stability and bread volume (Sobolewska et al., 2020; Gu et al., 2021). Split K application often outperforms a single basal application for these traits, which is likely because it ensures a more continuous K availability during critical growth stages, thereby supporting protein synthesis, gluten formation, and starch structure (Lu et al., 2014). The results of the present study are generally consistent with these findings. Across sites, split K treatments (T3 and T4) significantly increased dough formation time, dough stability time, protein content, wet gluten content, and sedimentation value compared with the no-K control (T1).

However, site-specific responses highlight the importance of initial soil K status. At the low-K Daolang site, even a single basal application (T2) under K1 improved several quality parameters compared with T1, although split applications provided additional benefits. This observation is consistent with that of Liu et al. (2026), who reported that basal K application can improve wheat quality under K-deficient conditions. In contrast, at the higher-K Wugong and Qianxian sites, T2 showed no significant advantage over T1 for protein content, wet gluten, or sedimentation value, whereas split K applications (particularly T4) produced clear improvements. This agrees with Lu et al. (2014), who demonstrated that split K is more effective in improving grain quality under conditions of adequate soil K supply. Similarly, dough tensile properties, including tensile area and maximum resistance to extension, responded most strongly to the three- split application (T4), with two-split treatments (T3) showing intermediate effects.

These patterns suggest that in K-deficient soils, basal K application alone can improve grain quality, whereas in soils with moderate to high K availability, split K application is required to sustain K supply during the reproductive and grain-filling stages (Lv et al., 2017). Variations among studies and sites may be attributed to differences in wheat genotypes, soil K status, climatic conditions, and K application strategies (Beres et al., 2020). Overall, the superior performance of split K application—particularly when applied at jointing and anthesis—highlights its importance for processing quality in modern production systems. Optimizing the timing of K fertilization therefore represents a practical strategy to enhance both grain yield and end-use quality, depending on local soil conditions.

### 4.5 Implications and limitations

The findings of this study have important implications for optimizing K management in winter wheat production systems. The results demonstrate that split K application, particularly with topdressing at the jointing and anthesis stages, can improve grain yield, quality, and KUE. In irrigated systems, these strategies can be effectively implemented through fertigation, which minimizes additional labor requirements while enhancing nutrient use efficiency. However, in rainfed or labor-constrained systems, multiple fertilizer applications may increase management complexity and operational costs and therefore require careful consideration of farm-level conditions. This study also has several limitations. The experimental duration was limited to two growing seasons, which may not fully capture long-term variability in environmental conditions and crop responses. Although the study included multiple sites with contrasting soil K levels, the results may still be influenced by site-specific factors such as soil type and climate. In addition, although different K application rates and timings were evaluated, a formal sensitivity analysis was not conducted, and future research incorporating such approaches would provide deeper insight into optimizing K management under varying environmental conditions. Furthermore, the study focused on selected K management strategies, and interactions with other agronomic practices were not explored. Therefore, longer-term and multi-environment studies are needed to further validate the sustainability and broader applicability of these findings.

## 5 Conclusion

This study evaluated the effects of K fertilizer application methods on soil K dynamics, plant uptake, yield formation, NEB, and grain quality of winter wheat in the Huang-Huai-Hai region. Both basal and split K applications increased the soil AK during early growth; however, split K application maintained higher surface soil K during later stages, particularly when applied at jointing and anthesis. Yield responses varied by site and soil K status. At the low-K Daolang site, K application increased the 1, 000-grain weight and grain yield, whereas at the higher-K Wugong and Qianxian sites, yield improvement was observed only under split K application. The highest yield and KUE were achieved with the three-stage split strategy (2:3:5 at sowing, jointing, and anthesis). Grain quality was not significantly affected by single basal K application but was improved by split K application, which enhanced protein content, wet gluten, sedimentation value, and dough rheological properties, which is likely due to improved post-anthesis dry matter accumulation. Based on the agronomic and economic results under the specific conditions of this study, an application rate of 120kg K_2_O ha^-1^ appears suitable for the moderately K-deficient eastern areas, while 60kg K_2_O ha^-1^ is more appropriate for the high-K western areas. However, these recommendations should be adapted according to local economic conditions and input–output relationships. Overall, split K application using a 2:3:5 ratio provides an effective strategy to optimize yield, KUE, and grain quality. However, as this study was conducted over two growing seasons, long-term sustainability and environmental impacts require further investigation.

## Data Availability

The original contributions presented in the study are included in the article/**Supplementary Material**, further inquiries can be directed to the corresponding author/s.
