# Supplementary material for: Impact of split application of potassium fertilizer on yield, quality, and economic benefits of winter wheat
Source: Front Plant Sci. 2026 May 13;17:1827899. doi: 10.3389/fpls.2026.1827899 (PMC13212479; doi:10.3389/fpls.2026.1827899)
Supplement: Supplementary file 1 [file Table1.docx]

**Supplementary File**

**Impact of split application of potassium fertilizer on yield, quality and economic benefits of winter wheat**

Shengyan Pang^1†^, Muhammad Fraz Ali^1†^, Xin Wang^1^, Yongbing Wang^1^, Xiaotian Ren^1^, Tahir Shah^2^, Xiang Lin^1^, Dong Wang^1,3*^

1. State Key Laboratory of Crop Stress Resistance and High-Efficiency Production, College of Agronomy, Northwest A & F University, Yangling 712100, Shaanxi, China
2. College of Natural Resources and Environment, Northwest A&F University, Yangling, Shaanxi 712100, China
3. Anhui Science and Technology University, Anhui 233100 Fengyang, China

***Correspondence Email:** [**wangd@nwafu.edu.cn**](mailto:wangd@nwafu.edu.cn)

**†:** These authors contributed equally to this work

**Table S1:** Analysis of variance for grain yield and its components under different potassium treatments across experimental sites in winter wheat

| Source of Variation | df | Spike Number | Grains per Spike | 1000-GW | Grain Yield |
| --- | --- | --- | --- | --- | --- |
| K method (T) | 4 | 4.19** | 10.09*** | 12.62*** | 311.42*** |
| Site (S) | 2 | 7.14** | 44.00*** | 91.25*** | 826.88*** |
| K rate (K) | 1 | 5.44* | 0.01ns | 0.56ns | 27.25*** |
| T × S | 8 | 0.70ns | 0.10ns | 0.21ns | 8.50*** |
| T × K | 4 | 0.68ns | 0.55ns | 0.02ns | 2.58* |
| S × K | 2 | 0.37ns | 0.09ns | 0.02ns | 8.62*** |
| T× S × K | 8 | 0.21ns | 0.24ns | 0.06ns | 1.76ns |

**Table S2:** Components of net economic benefits (NEB) under different potassium treatments across experimental sites in winter wheat

|  |  |  | **Price** | |
| --- | --- | --- | --- | --- |
| **Components** | **Items** | **Fertilizer Treatments** | **CNY kg^-1^** | **CNY ha^-1^** |
| Agricultural input | Fertilizer Cost | T1 | - | 2064 |
|  |  | K1T2 | - | 2640 |
|  |  | K1T3 | - | 2640 |
|  |  | K1T4 | - | 2640 |
|  |  | K1T5 | - | 3024 |
|  |  | K2T1 | - | 2208 |
|  |  | K2T2 | - | 2208 |
|  |  | K2T3 | - | 2208 |
|  |  | K2T4 | - | 2304 |
|  | Seed Cost | - | 3.0 | 450 |
|  | Pesticide Cost | - | - | 250 |
|  | Field service management | - | - | 1500 |
|  | Machinery Cost (Seeder + Harvesting) | - | - | 1500 |
| Agricultural Output | Local wheat sale price | - | 2.4 | - |

Note: The price of urea, Potassium (60% K2O), Controlled-release K fertilizer (44% K) and Triple superphosphate fertilizer (46% P2O5) was 2, 3.6, 3.6 and 3.5 CNY kg^-1^, respectively.

The net economic benefit was calculated based on grain yield and local market prices, while total production costs included fertilizer inputs, irrigation, and labor costs associated with fertilizer application. Although the economic results reflect local conditions during the study period, they provide a comparative assessment of treatment performance. It should be noted that economic outcomes may vary across regions due to differences in input costs and market prices.
